# Isolation of Extracellular Polymeric Substances from Biofilms of the Thermoacidophilic Archaeon *Sulfolobus acidocaldarius*

**DOI:** 10.3389/fbioe.2015.00123

**Published:** 2015-08-27

**Authors:** Silke Jachlewski, Witold D. Jachlewski, Uwe Linne, Christopher Bräsen, Jost Wingender, Bettina Siebers

**Affiliations:** ^1^Molecular Enzyme Technology and Biochemistry (MEB), Biofilm Centre, Centre for Water and Environmental Research (CWE), University Duisburg-Essen, Essen, Germany; ^2^Aquatic Microbiology, Biofilm Centre, Centre for Water and Environmental Research (CWE), University Duisburg-Essen, Essen, Germany; ^3^Core Facility for Mass Spectrometry and Elemental Analysis, Department of Chemistry and SYNMIKRO, Philipps-University of Marburg, Marburg, Germany

**Keywords:** Archaea, thermoacidophile, *Sulfolobus acidocaldarius*, biofilms, extracellular polymeric substances, proteins, enzymes

## Abstract

Extracellular polymeric substances (EPS) are the major structural and functional components of microbial biofilms. The aim of this study was to establish a method for EPS isolation from biofilms of the thermoacidophilic archaeon, *Sulfolobus acidocaldarius*, as a basis for EPS analysis. Biofilms of *S. acidocaldarius* were cultivated on the surface of gellan gum-solidified Brock medium at 78°C for 4 days. Five EPS extraction methods were compared, including shaking of biofilm suspensions in phosphate buffer, cation-exchange resin (CER) extraction, and stirring with addition of EDTA, crown ether, or NaOH. With respect to EPS yield, impact on cell viability, and compatibility with subsequent biochemical analysis, the CER extraction method was found to be the best suited isolation procedure resulting in the detection of carbohydrates and proteins as the major constituents and DNA as a minor component of the EPS. Culturability of CER-treated cells was not impaired. Analysis of the extracellular proteome using two-dimensional gel electrophoresis resulted in the detection of several hundreds of protein spots, mainly with molecular masses of 25–116 kDa and pI values of 5–8. Identification of proteins suggested a cytoplasmic origin for many of these proteins, possibly released via membrane vesicles or biofilm-inherent cell lysis during biofilm maturation. Functional analysis of EPS proteins, using fluorogenic substrates as well as zymography, demonstrated the activity of diverse enzyme classes, such as proteases, lipases, esterases, phosphatases, and glucosidases. In conclusion, the CER extraction method, as previously applied to bacterial biofilms, also represents a suitable method for isolation of water soluble EPS from the archaeal biofilms of *S. acidocaldarius*, allowing the investigation of composition and function of EPS components in these types of biofilms.

## Introduction

Microorganisms in the biofilm mode of life predominate, numerically and metabolically, in a wide variety of natural, technical, and medical environments (Costerton et al., 1995). Microbial biofilms are single- or multi-species communities that accumulate at interfaces (solid–liquid or solid–air), where the microorganisms live at high cell densities in a matrix of hydrated extracellular polymeric substances (EPS) (Hall-Stoodley et al., 2004). EPS are mainly polysaccharides, proteins, extracellular DNA (eDNA), and lipids. They mediate biofilm adhesion to surfaces and form a cohesive, three-dimensional polymer network interconnecting and immobilizing biofilm cells, and they provide mechanical stability to biofilms (Flemming and Wingender, 2010). The biofilm matrix acts as an external digestion system, keeping extracellular enzymes close to biofilm cells, capable of metabolizing dissolved, colloidal and solid biopolymers. The EPS matrix constitutes the immediate environment and conditions of life for biofilm organisms and thus, is regarded as a key component for the understanding of the biofilm mode of life. Bacterial biofilms have been intensively studied not only because of their ecological importance, application in biotechnology, and waste water treatment but also due to their potential role in human infections and function as environmental reservoirs for pathogens (Hall-Stoodley et al., 2004). Members of the Archaea have gained special research interest due to their adaptation to extreme environments. Though the potential of this domain especially for biotechnological applications of their enzymes is well documented, only recently fundamental studies about archaeal biofilms have been initiated (Schopf et al., 2008; Baker-Austin et al., 2010; Koerdt et al., 2010, 2012; Orell et al., 2013). It has become evident that environmental biofilm communities often contain both bacterial and archaeal species; in addition, laboratory investigations have shown that single archaeal species are able to form biofilms on diverse biotic and abiotic surfaces (Fröls, 2013; Orell et al., 2013).

Investigations of EPS involve sampling of biofilms, extraction, and in some cases, purification of EPS, and finally analysis of composition and identity of the EPS (Nielsen and Jahn, 1999; Denkhaus et al., 2007). The extraction efficiency for EPS varies depending on the origin, composition, and constituent microorganisms of the biofilm as well as on the extraction method used. There is no universal extraction method for the quantitative recovery of EPS from biofilms, and in some cases, complementary methods may have to be applied in order to obtain all fractions of the EPS matrix (Park and Novak, 2007). Different physical and chemical methods, including centrifugation, filtration, heating, blending, sonication as well as treatment with sodium hydroxide alone or in combination with formaldehyde, use of a complexing agent, or cation-exchange resin (CER), have been described for the extraction of EPS from monospecies biofilms and mixed population biofilms as well as flocs from natural environments and technical water systems (Nielsen and Jahn, 1999). Heating or addition of chemicals, such as sodium hydroxide and formaldehyde for the extraction process can result in the disruption of macromolecules, and chemicals like EDTA may also interfere with subsequent EPS analysis (Comte et al., 2006). In contrast, the CER method has been accepted as a mild EPS extraction method for many types of biofilms, causing limited cell lysis and no disruption and interference with EPS analysis (Sheng et al., 2010). Thus, an appropriate method has to be chosen and adapted to the properties of the biofilm under study with the aim to give an effective recovery of EPS, to cause minimal cell lysis, to avoid destruction of EPS, and to be compatible with EPS analysis methods.

Extracellular polymeric substances have often been studied in natural and laboratory biofilms of mixed-populations or single species grown under non-extreme conditions. There is some evidence that microbial biofilms in extreme environments also produce EPS as, for example, has been demonstrated for pellicle biofilms, composed of bacteria and archaea, from acid mine drainage solutions (pH 0.83–1.0), where extracted EPS components were found to be carbohydrates, proteins, DNA, and lipids (Jiao et al., 2010). Staining of monospecies archaeal biofilms with fluorescently labeled lectins and fluorescent DNA-binding dyes allowed the microscopic visualization of extracellular glycoconjugates (polysaccharides) and eDNA, indicating the presence of an EPS matrix. This was shown for archaeal extremophiles, including halophilic organisms (*Halobacterium*, *Haloferax*, and *Halorubrum* species; Fröls et al., 2012) and thermoacidophilic *Sulfolobus* species (Koerdt et al., 2010). However, detailed information on the composition of EPS from archaeal biofilms is still lacking, since EPS extraction and subsequent biochemical analysis have not been applied to these biofilms in contrast to the intensively studied EPS from biofilms of single bacterial species.

As outlined above, in Archaea – constituting the third domain of life with unique cellular and metabolic properties – the biofilm mode of life is evidently as ubiquitous and therefore comparably important as in Bacteria (Fröls, 2013). Although widely distributed in mesophilic habitats, most so far cultivable archaeal species are adapted to extremes of temperature, pH, salinity, or a combination thereof. With optimal growth requirements of 78°C and pH 2–3.5, the crenarchaeal members of the order Sulfolobales are adapted to both high temperature and acidic conditions, a property so far only found in Archaea but not in Bacteria. *Sulfolobus* spp. are easy to grow on minimal and complex media and several *Sulfolobus* genome sequences as well as comprehensive biochemical and functional genomics data are available (Zaparty and Siebers, 2011). *S. acidocaldarius* was first isolated from acid hot springs at Yellowstone National Park (Brock et al., 1972), and has become a well-established model strain for the archaeal domain. In contrast to the physiologically more versatile *S. solfataricus*, its genome size is 30% smaller and more genetically stable due to no mobile genetic elements. A robust and versatile genetic system is available for *S. acidocaldarius*, which enables the construction of in-frame markerless deletion mutants, ectopic integration of foreign DNA, and an effective homologous expression system (Wagner et al., 2012), which is important for future analyses on gene functions in biofilm formation. For biofilm investigations, the thermoacidophilic growth requirements represent a special challenge. The focus of the present study was to get first insights into the EPS composition of this archaeon by (i) cultivation of *S. acidocaldarius* as unsaturated biofilms yielding sufficient amounts for EPS isolation and analyses, (ii) by selecting a method suitable for EPS extraction from *S. acidocaldarius* biofilms, and (iii) by subsequent biochemical characterization of the isolated EPS.

## Materials and Methods

### Growth conditions

*S. acidocaldarius* DSM 639 was grown to the mid-exponential growth phase in liquid Brock medium (Brock et al., 1972) supplemented with 0.1% (w/v) N–Z-amine and 0.2% (w/v) dextrin at 78°C for 2 days with shaking (180 rpm) up to an optical density at 600 nm of 0.6–0.8. For biofilm cultivation, culture fluid was densely streaked in lines on plates of Brock medium (pH 3.5) solidified with gellan gum (6 g L^−1^; Gelzan™ CM, Sigma-Aldrich, Germany) and supplemented with 3 mM CaCl_2_ and 10 mM MgCl_2_. The plates were sealed in plastic bags and incubated at 78°C for 4 days.

### Characterization of biofilms

Determination of dry weight and residue on ignition as well as loss on ignition (volatile matter) of *S. acidocaldarius* DSM 639 biofilms was performed according to the standard DIN EN 12880 and DIN EN 12879, respectively. Briefly, samples of approximately 1 g (wet weight) biofilm mass were scraped from the surface of gellan gum plates after 4 days of incubation and successively dried to constant weight at 105°C and 550°C for determination of dry weight and residue on ignition, respectively. Loss on ignition was calculated by the difference between dry weight and residue on ignition values. For the determination of cation content, cells were disintegrated by acid digestion, using HNO_3_/H_2_O_2_, in combination with microwave treatment, and the cations were quantified by inductively coupled plasma optical emission spectrometry (ICP-OES) according to ISO 11885 (2007) at the IWW Water Centre (Mülheim an der Ruhr, Germany).

### Determination of total cell counts and colony counts

Total cell counts and colony counts were determined in biofilm suspensions. The total cell number was determined by staining with 4′,6-diamidino-2-phenylindole (DAPI; 25 μg mL^−1^ in 2% formaldehyde, 20 min) and enumeration at 1000-fold magnification, using an epifluorescence microscope. Viability of cells was determined in terms of colony forming units (CFU). Dilutions (1 mL) of the biofilm suspension as well as cells obtained after each EPS isolation procedure were spread onto gellan gum-solidified Brock medium for determination of the CFU after 4 days at 78°C. The impact of the phosphate buffer (pH 7.0) used for EPS isolation on cell viability was determined by comparing CFUs of cells suspended in either buffer or standard Brock medium (pH 3.5) under different conditions (shaking with/without CER). The statistical significance of differences between the CFUs was determined using a two-tailed paired Student’s *t-*test.

### EPS extraction

Biofilm mass was scraped from the surface of gellan gum-solidified Brock medium using a spatula, and suspended in phosphate buffer (2 mM Na_3_PO_4_ × 12 H_2_O, 4 mM NaH_2_PO_4_ × 1 H_2_O, 9 mM NaCl, 1 mM KCl, pH 7.0) at a concentration of 0.1 g wet weight/10 mL. For EPS extraction, five methods were applied: shaking, CER extraction, EDTA extraction, crown ether extraction, and NaOH extraction. For the shaking procedure, biofilm suspensions were transferred into 50 mL polypropylene centrifuge tubes in 10 mL aliquots and shaken at highest capacity for 20 min on a shaker (VortexGenie^®^2, Scientific Industries, USA). For CER extraction, biofilm suspensions were transferred into 50 mL centrifuge tubes in 10 mL aliquots. To each tube, 2 g of hydrated CER (Dowex^®^ Marathon^®^ C sodium form, Sigma-Aldrich, Germany) washed twice with phosphate buffer (15 min; 10 mL g^−1^ Dowex) were added. The samples were shaken at highest capacity for 20 min on a shaker (VortexGenie^®^2, Scientific Industries, USA). For EDTA extraction, 30 mL 2% (w/v) EDTA (disodium salt) solution in deionized water were added to 30 mL biofilm suspension, and the mixture was stirred for 3 h at 4°C. For crown ether extraction, 30 mL of biofilm suspension were centrifuged, the pellet was suspended in 30 mL crown ether solution (30 mM dicyclohexyl-18-crown-6-ether in 50 mM Tris buffer, pH 8.0), and the suspension was stirred for 3 h at 4°C (Wuertz et al., 2001). For NaOH extraction, 12 mL 1 M NaOH was added to 30 mL biofilm suspension and the mixture was stirred for 3 h at 4°C. After the respective treatment, the pH values of the extracts were determined and the samples were centrifuged at 20,000 × *g* for 20 min (4°C). The cells were resuspended in one volume of deionized water. The supernatants were filter sterilized (0.22 μm pore size) and the filtrates were dialyzed against deionized water (molecular weight cut-off 3,500 Da, 2× 1 h and overnight) to obtain high molecular weight compounds (EPS fraction).

### Chemical analysis of EPS

Carbohydrates and proteins were quantified in the biofilm suspensions as well as in the cell suspensions after separation from EPS, the filter sterilized supernatant (total extracellular material), and the EPS solution after dialysis. Carbohydrate concentrations were determined with the phenol sulfuric acid method using d-glucose as a standard (Dubois et al., 1956). For determination of protein concentrations, a modified Lowry assay was applied using commercial reagents (Sigma) and bovine serum albumin as a standard (Wingender et al., 2001). DNA was quantified using the Quant-iT PicoGreen dsDNA reagent kit (Invitrogen, Molecular Probes) and λ-DNA as a standard. The measurement was carried out with a fluorimeter (SFM 25, Kontron instruments) set to an excitation wavelength of 480 nm and emission readings at 520 nm.

### Two-dimensional gel electrophoresis

For two-dimensional gel electrophoresis (2 DE) analyses, Tris buffer (pH 8.0), MgCl_2_, and Benzonase (purity >99%; Novagen) to final concentrations of 50 mM, 10 mM, and 65 U mL^−1^, respectively, were added to the EPS solutions, and samples were incubated for 1 h at 37°C. Subsequent dialysis using Spectra/Por dialysis tubing (MWCO 12–14 kDa) was performed in three steps, each against 5 L deionized water, with the first two changes after 1 h and the final step overnight. Determination of protein content in the isolated EPS fractions was carried out according to a modified Lowry procedure (Peterson, 1977) and aliquots with a protein amount of 100–400 μg were lyophilized. For two-dimensional gel electrophoresis, lyophilized samples were first resuspended in 380 μL IEF buffer [7 M urea, 2 M thiourea, 4% (w/v) CHAPS, 5 mM tributylphosphine, 0.25% (w/v) Servalyt 3–10 ampholyte (Serva), a few crystals bromophenol blue prepared in ultrapure water; Ultrapur, Merck] and incubated at room temperature for 1 h. The samples were then applied to an IEF tray, an IPG strip was added (immobilized pH gradient with pH range of 3–10, linear or non-linear, Bio-Rad, Germany), and covered with mineral oil (3 mL). After overnight rehydration at 20°C, samples were subjected to IEF at a maximum of 75 μA in five steps at (i) 200 V (45 min), (ii) 500 V (45 min), (iii) 1,000 V (45 min), (iv) 10,000 V (4 h), and (v) finally at 10,000 V (5 h) using a Protean IEF cell (Bio-Rad, Germany). Afterwards, the strips were applied to the second dimension separation or stored at −20°C. For the second dimension, separation 20 cm × 20 cm 12% polyacrylamide gels were used. After polymerization (4 h), gels were stored at 4°C overnight. Following two 15 min equilibration steps [first step in 6 M urea, 30% (w/v) water free glycerol, 2% (w/v) SDS, 0.05 M resolving gel buffer, and 0.1 g dithiothreitol (DTT); second step in the same buffer containing 0.5 g iodoacetamide instead of DTT] focused IPG strips were washed with 1× electrophoresis buffer (Rotiphorese^®^ 10× SDS-PAGE, Roth) and applied to the SDS gel together with a wick soaked with 5 μL of Mark12, Invitrogen, or PageRuler™Plus Prestained Protein Ladder (Fermentas) and dried. Both IPG strip and marker wick were fixed using 2 mL 0.5% (w/v) agarose containing bromophenol blue. Using a Protean II Xi cell (Bio-Rad, Germany) with 1× SDS–Tris–glycine running buffer, electrophoresis was carried out at initially 20 mA for 45 min and subsequently at 35 mA for 4.5 h, and proteins were visualized by silver staining (Blum et al., 1987).

### Identification of EPS proteins via nanoRSLC-Orbitrap LC–MS/MS

Dialyzed EPS proteins were analyzed in the Core Facility for Mass Spectrometry at the Chemistry department of the Philipps-University Marburg (Germany). Samples were digested by the addition of Sequencing Grade Modified Trypsin (Promega) and incubated at 37°C overnight. The mass spectrometric analysis of the samples was performed using an Orbitrap Velos Pro mass spectrometer (ThermoScientific). An Ultimate nanoRSLC system (Dionex), equipped with a custom 20 cm × 75 μm C18 RP column filled with 1.7 μm beads, was connected online to the mass spectrometer through a Proxeon nanospray source. A total of 1–15 μL of the tryptic digest (depending on sample concentration) were injected onto a C18 pre-concentration column. Automated trapping and desalting of the sample was performed at a flowrate of 6 μL/min using water/0.05% formic acid as solvent. Separation of the tryptic peptides was achieved with the following gradient of water/0.05% formic acid (solvent A) and 80% acetonitrile/0.045% formic acid (solvent B) at a flow rate of 300 nL/min: holding 4% solvent B for 5 min, followed by a linear gradient to 45% solvent B within 30 min and linear increase to 95% solvent B in additional 5 min. The column was connected to a stainless steel nanoemitter (Proxeon, Denmark) and the eluent was sprayed directly toward the heated capillary of the mass spectrometer using a potential of 2300 V. A survey scan with a resolution of 60,000 within the Orbitrap mass analyzer was combined with 10 data-dependent MS/MS scans with dynamic exclusion for 30 s using HCD combined with orbitrap detection at a resolution of 7,500. Data analysis was performed using Proteome Discoverer 1.4 (ThermoScientific) with SEQUEST and MASCOT (version 2.2; Matrix science) search engines using either SwissProt or NCBI databases.

Only proteins with two or more unique identified peptides were taken as significant. The identified proteins were further analyzed and functionally categorized bioinformatically using the arCOG database (archaeal cluster of orthologous groups of proteins) and the updated *S. acidocaldarius* genome annotation (Esser et al., 2011). The subcellular localization was predicted by PSORTb (Yu et al., 2010).

### Fluorimetric assays of extracellular enzyme activities

The pH of the cell-free, dialyzed EPS solutions were adjusted to pH 3.5 (corresponding to the pH value of the biofilm growth medium) and screened for enzyme activities of seven different enzyme groups. The fluorogenic 4-methoxy-β-naphthylamide and methylumbelliferyl (MUF) substrate analogs (Sigma-Aldrich, Germany) were used as 2 mM stock solutions in 2-methoxyethanol, i.e. l-alanine-4-methoxy-β-naphthylamide, 4-MUF-α-d-glucopyranostide, 4-MUF-β-d-glucopyranoside, 4-MUF-*N*-acetyl-β-d-glucosaminide, 4-MUF-stearate, 4-MUF-butyrate, and 4-MUF-phosphate. A total of 190 μL of the isolated EPS fractions were supplemented with 10 μL of the respective substrate stock solutions. A total of 10 μL 2-methoxyethanol was used as negative control. Incubation was carried out in 96-well plates at 70°C. Before fluorescence measurements, plates were agitated using the shaking function of the plate reader at 1.5 mm amplitude for 5 s. Fluorescence was determined using the Infinite Pro 200 microplate reader (Tecan) with 360 nm and 450 nm (MUF substrates) as well as 330 nm and 420 nm (4-methoxy-β-naphthylamide) as emission and excitation wavelength, respectively.

Non-enzymatic substrate hydrolysis was measured mixing 10 μL of the respective substrate solution with 190 μL phosphate buffer pH 3.5 (without isolated EPS) as negative controls. 4-methylumbelliferone (0–200 mM) and 4-methoxy-β-naphthylamide (0–100 mM) were utilized for calibration.

### Zymographic analysis of protease and esterase activity

For one dimensional zymographic analyses native, SDS free discontinuous 10% polyacrylamide gels supplemented with 0.1% casein (C3400 Sigma-Aldrich, Germany) or gelatin (48723 Sigma-Aldrich, Germany) were used. EPS samples (5 μg protein) were supplemented with non-reducing Roti^®^-Load 2 (4×) sample buffer (Roth, Germany). After electrophoresis [125 V, 90 min, Rotiphorese SDS running buffer (Roth, Germany)], gels were washed twice (30 min) in Novex^®^ Zymogram renaturing buffer (Invitrogen) and incubated in developing buffer pH 3.8 or 7.0 (Novex^®^ Zymogram, Invitrogen) overnight at 37°C or 70°C with one buffer exchange after 60 min. Protease activity was detected as halo formation upon Simply Blue Safe Stain (Invitrogen) for 1 h and subsequent destaining in water (2× for 1 h). Imaging was carried out with the Bio-Rad Densitometer GS-700. For 2D zymographic analyses, gels were prepared as described above omitting SDS and adding casein. Samples corresponding to a total protein amount of 400 μg were applied. Washing (2× 1 h), developing (1× 1 h, 1× 48 h), and staining (1× 2 h) were performed using the same buffers as for the 1 DE analyses. 2 DE gels were also analyzed for esterase activity. Therefore, gels were washed in 100 mM Tris–HCl, pH 8, 25% (v/v) isopropanol (2× 30 min) and incubated in 100 mL 5 mM 4-methylumbellyferyl-butyrate (Sigma, in renaturing buffer) at pH 3.0 and 8.0, respectively (10 min). The Molecular Imager Gel Doc XR System (Bio-Rad) was used for visualization.

## Results

### Composition of *S. acidocaldarius* biofilm

Biofilms of *S. acidocaldarius* grown at 78°C for 4 days on gellan gum-solidified Brock medium contained a total biomass of approximately 0.6 g (wet weight) per Petri dish (100 mm in diameter). The dry weight of these biofilms accounted for 24.9 ± 1.4% (0.249 ± 0.014 mg/g wet weight; *n* = 3), of which approximately 97% (0.242 ± 0.013 mg/g wet weight) and 3% (0.008 ± 0.002 mg/g biofilm wet weight) were organic and inorganic matter, respectively, as determined by loss of ignition and residue on ignition. The total cell count was 1.2 ± 0.4 × 10^12^ per gram of biofilm wet weight (*n* = 3). As major multivalent cations in the biofilms, magnesium > iron > calcium > copper (in decreasing order) were observed (Table 1).

**Table 1:** **Concentration of multivalent cations in *S. acidocaldarius* biofilms cultivated on Brock medium plates at 78°C for 4 days**.

Cation	Concentration (μg g^−1^ biofilm wet weight)
Mg^2+^	385.0 ± 7.1
Fe^2+/3+^	162.0 ± 56.6
Ca^2+^	125.0 ± 7.1
Cu^1+/2+^	5.6 ± 0.8
Sr^2+^	0.265 ± 0.064
Zn^2+^	0.235 ± 0.007
Al^3+^	0.147 ± 0.095
Ba^2+^	0.039 ± 0.004
B^3+^	0.037 ± 0.001
Mo^4+/6+^	0.031 ± 0.001
Cr^3+/6+^	0.004 ± 0.001
Co^2+/3+^	0.003 ± 0;

*Cations were analyzed by ICP-OES*.

## Efficiency of EPS Extraction Procedures

**Figure 1:**
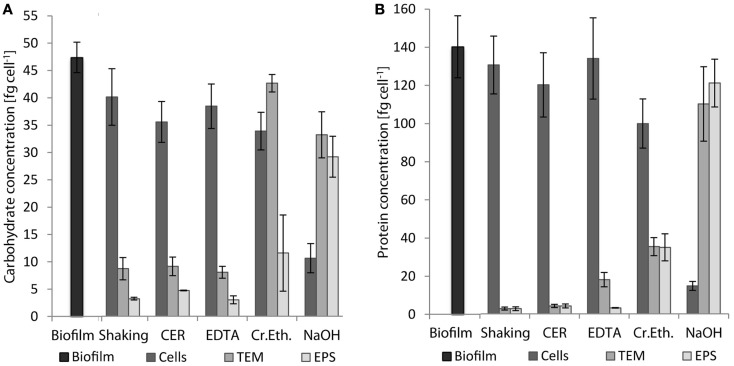
**Concentrations of carbohydrates (A) and proteins (B) in whole biofilms of *S. acidocaldarius* and different biofilm fractions after EPS extraction with shaking, CER, crown ether (Cr.Eth.), EDTA, or NaOH**. EPS were obtained from biofilms recovered from Brock medium plate cultures after incubation for 4 days at 78°C (*n* = 3). TEM, total extracellular material.

Extracellular polymeric substances were isolated from biofilms of *S. acidocaldarius* scraped from the medium plates using five different procedures, including shaking, shaking in the presence of CER, stirring with added NaOH, EDTA, and crown ether, respectively. Cells were separated from the extracellular material by centrifugation and the cell-free supernatant was dialyzed against deionized water to obtain the EPS. The different fractions (biofilm suspensions, cells after separation from extracellular material, total extracellular material, EPS solution) were analyzed for total carbohydrates, proteins, and DNA. Independent of the extraction method, the EPS preparations were always found to contain carbohydrates, proteins, and minor amounts of DNA (Figure 1; Table 2).

**Table 2:** **Carbohydrate, protein, and DNA content of EPS extracted from *S. acidocaldarius* biofilms with shaking, CER, EDTA, crown ether, and NaOH**.

Extraction method	Carbohydrates		Proteins		Protein to carbohydrate ratio	DNA
	(fg cell^−1^)	(%)^a^	(fg cell^−1^)	(%)^a^		(fg cell^−1^)
Shaking	3.2 ± 0.3	6.7	2.7 ± 0.9	2.0	0.86	0.09 ± 0.02
CER	4.7 ± 0.1	9.8	4.2 ± 1.1	3.0	0.90	0.91 ± 0.48
EDTA	3.0 ± 0.7	6.2	3.2 ± 0.2	2.3	1.07	0.40 ± 0.29
Crown ether	11.4 ± 6.9	24.2	35.2 ± 7.2	25.1	3.07	1.50 ± 0.34
NaOH	28.9 ± 3.7	61.0	122.0 ± 12.6	87.0	4.22	0.99 ± 0.29

*^a^Percentage of carbohydrate or protein fraction with respect to total biofilm (*n* = 3)*.

Shaking, CER, and EDTA extraction resulted in similar carbohydrate content in the analyzed fractions, i.e., cell fraction (35–40 fg/cell), total extracellular material (8–9 fg/cell), and EPS fraction (3–4.7 fg/cell). The protein concentrations of 121–141 fg/cell in the cell fraction, 2.8–18 fg/cell in the total extracellular material, and 2.7–4.2 fg/cell in the EPS were also similar between these three isolation methods.

The CER method tended to yield the highest EPS carbohydrate and protein concentrations among the three methods. Preliminary analysis of CER-extracted EPS by acid hydrolysis and thin-layer chromatography revealed d-glucose as the main neutral carbohydrate component (data not shown).

The highest concentrations of carbohydrates and proteins in the EPS were observed after crown ether (carbohydrates 11.4 fg/cell, proteins 35.3 fg/cell) and NaOH (carbohydrates 29 fg/cell, proteins 122 fg/cell) extraction, respectively. However, crown ether treatment appeared to interfere with the analytical measurements as indicated by the inconsistent amount of carbohydrates determined for the total biofilm, which is exceeded by the sum of the total extracellular carbohydrates and the carbohydrate content of the cell fraction by 60%. Also, the EPS analysis after NaOH extraction appeared inaccurate because of the high discrepancy between carbohydrate (29 fg/cell) and protein (122 fg/cell) content of the EPS compared to the cell fraction (carbohydrate 10.5 fg/cell, protein 14.8 fg/cell), which suggests cell damage or lysis during preparation. This was also visually observed as transition of the suspended biofilms from brownish/turbid to clear/viscous upon NaOH treatment.

In addition to carbohydrates and proteins, 0.09–1.5 fg/cell DNA was detected in the EPS fraction with highest amounts for crown ether followed by NaOH, CER, and shaking in the presence of EDTA (Table 2). The protein to carbohydrate mass ratio of the total biofilm of *S. acidocaldarius* was determined to be 2.96. Only for crown ether extraction, a similar value was obtained (3.07) in EPS. All other EPS isolation methods resulted in different protein/carbohydrate ratio with a slightly higher carbohydrate content in the shaking (0.86) and CER (0.90) isolation, whereas the protein and carbohydrate content was the same in the EDTA prepared EPS (1.07). Significantly, more proteins than carbohydrates were observed in the NaOH extracted EPS (4.22; Table 2).

Determination of the pH in the solutions after EPS extraction revealed a constant neutral pH for shaking and CER and a slightly acidic pH for EDTA (5.14 ± 0.03). pH values in the alkaline range were observed for NaOH and crown ether with values of 13.28 ± 0.13 and 8.12 ± 0.07, respectively.

### Effect of EPS extraction on total and viable cell counts

**Figure 2:**
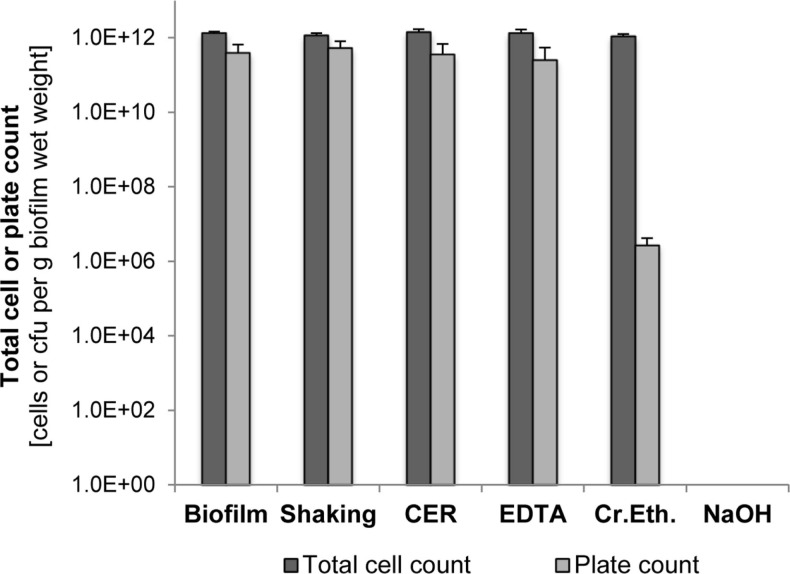
**Total cell counts and plate counts of *S. acidocaldarius* after EPS extraction using shaking, CER, NaOH, crown ether (Cr.Eth.), and NaOH**. Measurements were performed using suspensions of intact biofilm and treated cells in phosphate buffer (pH 7.0). Total cell counts were determined via counting of DAPI-stained cells. Colonies formed on Brock medium plates were counted after incubation for 4 days at 78°C (*n* = 3).

The impact of the EPS isolation method on cell integrity was analyzed by determining the total and viable cell counts after isolation and comparison to the biofilm cells before isolation. As shown in Figure 2, shaking, CER, and EDTA had nearly no effect on both total and viable cell counts compared to the untreated suspended biofilm cells. Culturable cells and total cell counts were in the same order of magnitude. However, the crown ether method resulted in a significant loss of culturability, whereas the total cell count was not changed. Use of NaOH caused a complete loss of total cells below the detection limit as well as of culturability.

The pH value of the isolation medium for biofilm suspension before EPS extraction, either phosphate buffer pH 7.0 or Brock medium pH 3.5, did not significantly influence the culturability (*p* > 0.05, two-tailed paired Student’s *t*-test). Also, the additional stirring (4°C, 3 h) under both pH conditions in the NaOH, EDTA, and crown ether isolation methods was not harmful for the culturability, indicating that even for the thermoacidophilic *S. acidocaldarius*, a pH 7.0 is suitable in the EPS isolation procedure.

### Electrophoretic analysis of EPS proteins

**Figure 3:**
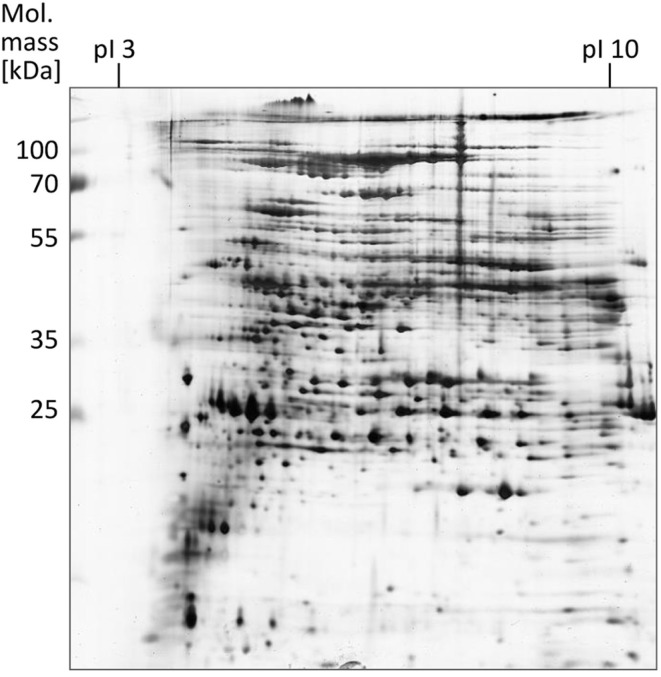
**EPS proteins of *S. acidocaldarius* biofilms separated by 2 DE and visualized by silver staining**. EPS were isolated from biofilms collected from Brock medium plates after 4 days of incubation at 78°C. A total of 200 μg EPS protein were applied for IEF (non-linear IPG strips). Molecular mass marker: PageRuler Prestained Protein Ladder (Fermentas).

The proteins obtained after EPS extraction using the different methods were analyzed by 2 DE using linear IPG strips and visualization by silver staining (Supplementary Material; Figure 1). Most protein spots (approximately 600) were obtained after crown ether isolation, followed by CER (~500 spots) and shaking (~300 spots). Utilization of NaOH for EPS extraction led to poor resolution of the protein spots particularly in the low molecular weight range. With EDTA, no separation could be achieved at all presumably due to elevated electrical resistance and accompanying high voltage during IEF. With application of linear IPG strips, the majority of proteins observed had a molecular mass between 25 and 115 kDa and a isolelectric point between 5 and 8. A refined analysis using non-linear IPG strips with an enhanced resolution in the pI range of 4–7 carried out with EPS proteins obtained by the CER method led to an increased number of protein spots (~1000) compared to the linear IPG strip separation (Figure 3).

### Identification of EPS proteins via nanoRSLC-Orbitrap LC-MS/MS

**Figure 4:**
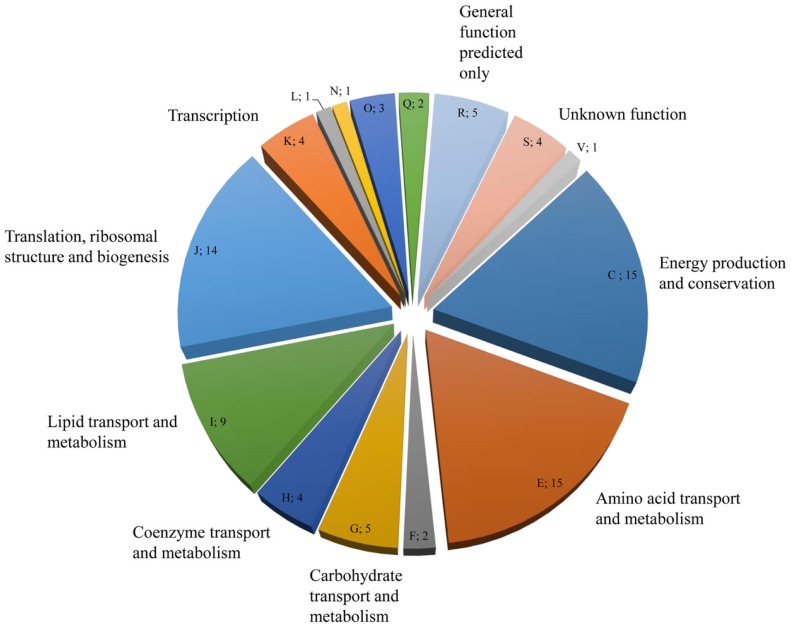
**Distribution and number of *S. acidocaldarius* biofilm EPS proteins in the different arCOGs functional categories**. For identification, EPS isolated from unsaturated *S. acidocaldarius* biofilms (4 days, 78°C) was digested with trypsin and applied for nanoRSLC-Orbitrap LC-MS/MS analysis. Proteins were assigned to arCOG functional categories (capital letters). F, Nucleotide transport and metabolism, L: Replication, recombination, and repair, N: Cell motility, Q: Secondary metabolites biosynthesis, transport, and catabolism, V: Defense mechanism.

NanoRSLC-Orbitrap LC–MS/MS analyses of the EPS proteins after tryptic digestion gave first insights into the extracellular proteome and resulted in the identification of 85 proteins, which could be assigned to 15 functional arCOG categories. (Figure 4; Table S1 in Supplementary Material). The majority of proteins (53) were categorized into the four arCOG clusters energy production and conversion (arCOG C), amino acid transport and metabolism (arCOG E), lipid transport and metabolism (arCOG I), and translation, ribosomal structure, and biogenesis (arCOG J). Most proteins (73) were predicted to be of intracellular localization, one, a putative ATPase involved in archaella (archaeal flagella) biosynthesis, is associated with the cytoplasmatic membrane and for the remaining 11 proteins no subcellular localization could be assigned. However, none of the identified proteins possessed a predictable signal peptide for secretion.

### Enzyme activities

**Figure 5:**
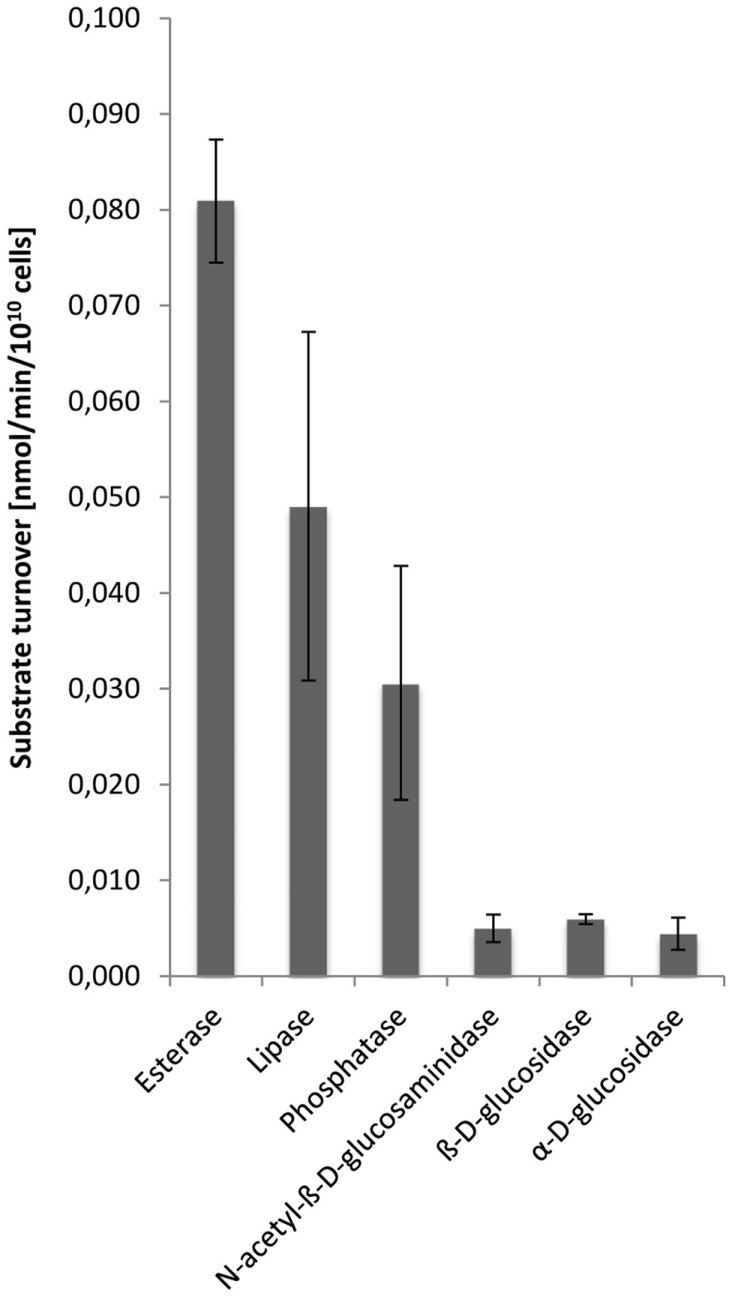
**Enzyme activities in the EPS from *S. acidocaldarius* biofilms**. EPS were extracted with the CER method from unsaturated biofilms collected from Brock medium plates after 4 days of incubation at 78°C. Enzyme activities in EPS solutions were determined at pH 3.5, using a microtiter plate assay as described in the Section “Materials and Methods.”

Hydrolytic enzyme activity was determined in cell-free dialyzed EPS solutions at pH 3.5 corresponding to the pH value of the growth medium, and thus should reflect the external environment of extracellular enzymes in the *S. acidocaldarius* biofilms. Esterases were demonstrated to be most active followed by lipases, phosphatases, *N*-acetyl-β-d-glucosaminidases, β-d-glucosidases, and α-d-glucosidases, while peptidase activity was not observed (Figure 5).

**Figure 6:**
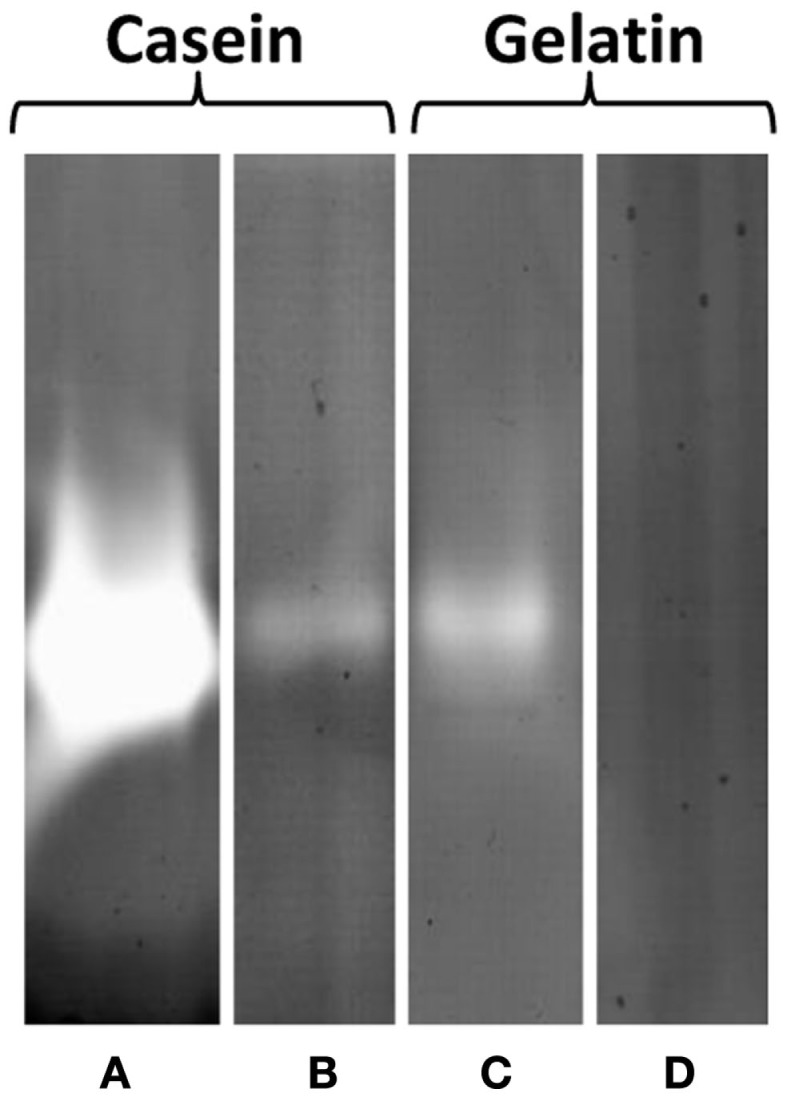
**Protease activity of EPS prepared from *S. acidocaldarius* biofilms, visualized in zymogram gels**. EPS (5 μg protein/lane) were electrophoresed in gels containing 0.1% casein or 0.1% gelatin and incubated at either pH 3.8 **(A,C)** or 7.8 **(B,D)** for 24 h at 78°C. EPS were isolated with the CER method from biofilms collected from Brock medium plates after 4 days of incubation at 78°C. Molecular mass marker: PageRuler Prestained Plus Protein Ladder (Fermentas).

However, in zymogram gels, protease activity could clearly be detected as single halo bands corresponding to a molecular mass of 47 kDa in EPS samples (5 μg protein) with casein and gelatin, respectively, as a substrate. Protease activity was higher at pH 3.8 than at 7.8 and casein was more actively degraded than gelatin as indicated by the band intensities (Figure 6). These results could be confirmed by 2 DE zymography with casein as substrate. The protein spot detected at pH 3.8 and less pronounced also at 7.8, corresponded to a molecular mass of 47 kDa and pI of 3–4. In 2 DE zymogram gels also, two weaker spots with lower molecular mass (27 kDa) and higher pI could be found. The observed 47 kDa signal in the 1 DE zymography showed pronounced heat stability at 100°C for 1 h. Preincubation of the EPS solution under these conditions did not result in a significant fading of the corresponding band. However, autoclaving at 121°C for 20 min resulted in a complete loss of activity under both pH conditions. Protease inhibitors (23 mM AEBSF, 2 mM bestatin, 100 mM EDTA, 0.3 mM E-64, 0.3 mM pepstatin A, Sigma-Aldrich) had nearly no effect on activity.

Also, the presence of esterases showing the major activities in the EPS fraction (see above) could be confirmed by 2 DE with MUF-butyrate as substrate at both pH 3.5 and 8.0. A spot cluster was detected corresponding to pI 3 and 28 kDa as well as two further signals at 20 and 35 kDa, respectively, and higher pI. Only at pH 8.0, an additional esterase signal was obtained at pI 10 and 25 kDa.

## Discussion

### Efficiency of EPS isolation methods

For the quantitative and qualitative EPS analyses, an appropriate cultivation technique of the thermoacidophilic Crenarchaeon *S. acidocaldarius* was required capable of high biofilm yields enabling the isolation of sufficient EPS amounts. However, so far only limited information about the cultivation of *Sulfolobus* spp. biofilms was available. In previous studies, *Sulfolobus* biofilms were grown on surfaces of glass slides, carbon-coated grids, μ-dishes or polystyrene microplates, and Petri dishes with the focus on the investigation of biofilm formation and analysis of attached biofilms with microscopic techniques, such as scanning electron microscopy or confocal laser scanning microscopy (Koerdt et al., 2010; Zolghadr et al., 2010; Henche et al., 2012). For quantitative and qualitative EPS analysis, destructive methods are usually employed, including dispersal of biofilm cells and extraction of EPS by physical and/or chemical procedures (Nielsen and Jahn, 1999). So far, the production of elevated biofilm mass on solid surfaces necessary for the recovery and subsequent biochemical analysis of EPS from *Sulfolobus* biofilms has never been reported. In the current study, *S. acidocaldarius* was grown as an unsaturated biofilm on the surface of gellan gum-solidified Brock medium plates, resulting in the reproducible formation of sufficient biomass suitable for subsequent EPS isolation and analysis. A similar approach proved useful for the cultivation of unsaturated bacterial biofilms grown on the surface of agar-solidified media with substantial production of EPS as has been shown, e.g., for *Pseudomonas aeruginosa* (Wingender et al., 2001) and *Pseudomonas putida* (Metzger et al., 2009).

For EPS analyses, the establishment, evaluation, and choice of suitable EPS isolation procedures and conditions are crucial, since all components should be obtained as entirely as possible without damaging the cells yielding to contaminations of the EPS extracts with intracellular components. Previous studies showed that there is no universal method applicable and possibly several methods are required to obtain all different EPS fractions (Park and Novak, 2007). As shown by Aguilera et al. (2008) by comparison of five EPS extraction methods applied to benthic eukaryotic biofilms from an acidic river, different methods potentially resulted in divergent yields of EPS and heavy metals. They also showed that most efficient EPS isolation, in that case with NaCl, resulted in highest contamination with the exclusively intracellular enzyme glucose-6-phosphate-dehydrogenase (G6PDH), indicating cell lysis. This again highlights the importance also known from other reports to carefully balance both EPS yield and cell damage. Thus, appropriate methods to detect cell lysis like protein/carbohydrate ratios and eDNA are most important in the establishment of EPS extraction methods. However, it is well established that eDNA can be a crucial component of the EPS matrix of several microorganisms and sometimes even be required for initial cell attachment and biofilm formation (Whitchurch et al., 2002; Flemming and Wingender, 2010; Jakubovics et al., 2013). Moreover, the ratio of proteins to carbohydrates can vary significantly between EPS of different microorganisms or different growth conditions and does not necessarily indicate cell lysis. The activity screening of strictly intracellular enzymes, like G6PDH for bacteria, is not applicable for EPS isolations with chemicals inhibiting enzyme activity like EDTA, which complexes cations, or NaOH and crown ether, which significantly increase the pH value, thus denaturing proteins (Frølund et al., 1996). The application of viability stains, for instance, using the Live/Dead staining method, is common to detect membrane disintegration (Wu and Xi, 2009). This method, however, could so far not be adapted to the type of biofilms used here for the archaeon, *S. acidocaldarius*. In this study, cell lysis was excluded by monitoring the impact on total and viable cells counts. Total (DAPI-stained, and thus, DNA-containing) cell counts and colony counts indicated that *S. acidocaldarius* cells agitated in the presence or absence of CER at pH 3.5 (Brock medium) or 7.0 (phosphate buffer) are largely insensitive toward mechanical shear forces and pH stress.

After establishing efficient culturing of *S. acidocaldarius* biofilms on solidified Brock medium plates (4 d, 78°C), in this study, five different EPS extraction procedures were analyzed and the influence on cell viability, EPS yield, and composition was determined. First, biofilms were suspended in phosphate buffer (biofilm suspension) followed by the actual EPS extraction. The five applied EPS isolation methods included shaking used as a simple reference method, shaking with the addition of CER as well as treatment with EDTA, crown ether, and NaOH. Finally, after removal of cells by centrifugation, subsequent filter sterilization, and dialysis of the supernatant, the final cell-free EPS solution was obtained.

Application of all five extraction methods provided EPS that contained carbohydrates, proteins, and minor amounts of DNA. However, the EPS yield and the ratio of the EPS components were strongly dependent on the extraction procedure. Thus, these archaeal EPS from *Sulfolobus* biofilms resemble the common situation for bacterial biofilms, where carbohydrates, proteins, and DNA are consistently found, with varying extraction efficiencies obtained when different isolation methods were compared (Jahn and Nielsen, 1995; Comte et al., 2006; Park and Novak, 2007; Aguilera et al., 2008). Comparison of the yield of total EPS in terms of carbohydrate, protein, and DNA content obtained by the five extraction methods applied in this study revealed the following order NaOH > crown ether > CER > EDTA > shaking. In agreement with this, the 2 DE analyses of the extracellular proteins showed the fewest proteins (approximately 300 spots) in the EPS isolated by shaking and no reduction of cell viability was observed. Hence, shaking represents a gentle physical procedure that isolates only not tightly bound, soluble EPS components without cell lysis, and effects on further EPS analysis.

Addition of CER improved the EPS yield via shaking. The stabilizing effect of multivalent cations, permitting electrostatic crosslinking interactions between polysaccharides and/or proteins, on the EPS is already known. The CER extraction procedure acts partly mechanically due to shear forces and partly chemically by the removal of divalent cations, with the destabilizing of the EPS matrix and the release of water soluble EPS (Jahn and Nielsen, 1995; Frølund et al., 1996). In this study, substantial removal of calcium and magnesium ions was confirmed for the *S. acidocaldarius* biofilms, showing an abstraction of at least 60% for magnesium to 80% for calcium from the biofilms (data not shown), similar to the values stated by Park and Novak (2007) for CER extraction of EPS from activated sludge. No removal of other cations was observed, such as iron and copper (this study) and iron and aluminum in the study of Park and Novak (2007). This observation indicates that the CER procedure seems to be selective for targeting calcium and magnesium ions and their associated EPS within the biofilm matrix. CER isolation of the EPS did not affect viability of *S. acidocaldarius* cells indicating that intracellular or membrane-derived contaminations in course of the isolation procedure are negligible. This is in accordance with studies on EPS isolation from bacterial biofilms, which also demonstrated that CER is suitable for mild extraction without elevated cell damage (Frølund et al., 1996; Chen et al., 2013).

However, both NaOH and crown ether led to substantial loss of culturability, probably due to cell lysis. Compared to the physical methods, the chemicals used for isolation remained in the EPS solution and interfered with certain subsequent analyses. EDTA interferes with protein determination according to the Lowry method by chelating copper ions and also with the isoelectric focusing applied in the 2 DE procedure. Crown ether interfered with the carbohydrate assay leading to unrealistic high concentrations. NaOH led to the loss of culturability and the reduction of total cell counts to below the detection limit, probably caused by the elevated pH resulting in cell lysis and denaturation of proteins, thus precluding any enzyme assays. In contrast to this, an advantage of CER apart from the low costs and easy handling is its complete removal from the EPS via centrifugation or settling of the resin while the chemical methods remain in the EPS and have to be removed via dialysis if possible.

### Analysis of EPS protein

The analysis of EPS proteins via 2 DE revealed an unexpected large number of proteins and first assays using fluorogenic substrates as well as zymography showed activities of diverse classes of hydrolytic enzymes, such as proteases, lipases, esterases, phosphatases, and glucosidases usually involved in extracellular degradation of polymers into assimilable mono- or oligomers (for review, see Wingender and Jaeger, 2002). A genomic analysis in *S. solfataricus*, a close relative of *S. acidocaldarius*, identified a total of more than 4% (corresponding to >100 proteins) of all encoded proteins in the genome to contain a signal sequence for protein export among them, e.g., the protease thermopsin and several endoglucanases (Albers and Driessen, 2002). In *Sulfolobus* species, few enzymes have previously been reported to be extracellular including the thermoacidophilic protease thermopsin (Lin and Tang, 1990, 1995; Rawlings, 2013), a carboxylesterase (Huddleston et al., 1995) and several endoglucanases (Limauro et al., 2001; Girfoglio et al., 2012), which coincides well with the respective enzyme activities reported here for the biofilms of *S. acidocaldarius*. Furthermore, the zymography signal of the protease observed in the EPS corresponded well to the molecular mass and pI reported for the glycosylated form of the thermopsin in *S. acidocaldarius*. However, in contrast to the enzyme activity assays, in the mass spectrometry analysis of the EPS fraction, none of these extracellular proteins could be identified and all of the proteins found were predicted to be cytoplasmic not harboring any predictable signal peptide sequence for secretion. Furthermore, the enzyme activities found could not be clearly correlated to detectable protein spots or bands in the gels, indicating that the enzyme amount present in the EPS allows for activity measurement, but is not sufficient for protein detection. Also, in the extracellular proteome of planktonic cell cultures of *Sulfolobus*, none of the known extracellular enzymes could be identified by SDS-PAGE separation and subsequent MS analyses of the excised bands. It has been concluded that these proteins are excreted directly to the medium, but only in very small quantities, not detectable by electrophoretic methods (Ellen et al., 2010a). The high number (several hundred) of proteins in the 2 DE gels was unexpected, since such high numbers of proteins were not identified in supernatants of planktonic cell cultures of *Sulfolobus* spp. (Ellen et al., 2010a). However, in contrast to the EPS proteins, most of the proteins isolated from culture supernatants contained a signal peptide for excretion. These signal peptide containing proteins were mainly argued to remain associated with the cell membrane, e.g., through C-terminal membrane anchors and only a small number appears to be directly released into the medium. Also, the S-layer appears to operate as a barrier for protein secretion. Accordingly, only limited protein secretion into the medium has been proposed for *Sulfolobus* spp. (Ellen et al., 2010a). The high number of cytoplasmic proteins in the EPS could be excluded to occur due to cell disruption caused by the EPS isolation procedure (no decrease of plate counts), and thus points either to membrane vesicles or to biofilm-inherent cell lysis: *Sulfolobus* cells were shown to form membrane vesicles involving an endosomal sorting complex required for transport (ESCRT) III-dependent budding process similar to the endosomal sorting pathway of exosomes in eukaryotes (Ellen et al., 2010b). For *S. islandicus*, growth inhibition of other *Sulfolobus* spp. by a protein factor isolated from these vesicles was demonstrated (Prangishvili et al., 2000). Membrane vesicle structures are also known from several bacteria and have been described in planktonic cultures, but also as a component of the matrices of biofilms (e.g., *P. aeruginosa*) (Schooling and Beveridge, 2006), where a role of these vesicles in antimicrobial defense, quorum sensing, providing hydrolytic activity, and stress response has been discussed. In the EPS isolation procedure reported here, such membrane vesicles may retain in the preparation, thus causing elevated numbers of cytoplasmic proteins. This is supported by the finding that 6 out of 32 proteins found in membrane vesicle proteomes from *Sulfolobus* spp. (Ellen et al., 2009) were also identified in the EPS. On the other hand, cell lysis within biofilms is established as inherent element of biofilm development in Bacteria (Jakubovics et al., 2013) and may also contribute to the large amount of cytoplasmic proteins in the EPS fraction. For bacterial biofilms, a major function of cell lysis in DNA exchange and nutrition has been discussed. However, in order to elucidate the reason for cytoplasmic protein enrichment in the matrix of *S. acidocaldarius* biofilms, further studies are required.

## Conclusion

In this study, a suitable and efficient method for the EPS extraction from *S. acidocaldarius* biofilms was achieved. Evaluation of the yield of each method together with the suitability for further analysis showed that CER is superior to the other applied methods leading to high EPS yields with no apparent cell lysis and no impact on subsequent biochemical analysis. The main component of *S. acidocaldarius* EPS was carbohydrates followed by proteins and DNA. Determination of the culturability for the different methods at a wide pH range demonstrated the high tolerance of the thermoacidophilic *S. acidocaldarius* toward pH stress at low temperature. Significant hydrolytic enzyme activity was observed in the EPS fraction. The high number of cytoplasmic proteins in the EPS fraction points to either the formation of extracellular membrane vesicles or cell lysis during biofilm development.

## Conflict of Interest Statement

The authors declare that the research was conducted in the absence of any commercial or financial relationships that could be construed as a potential conflict of interest.

## Supplementary Material

The Supplementary Material for this article can be found online at http://journal.frontiersin.org/article/10.3389/fbioe.2015.00123
