# Supplementary material for: Isolation of Extracellular Polymeric Substances from Biofilms of the Thermoacidophilic Archaeon Sulfolobus acidocaldarius
Source: Front Bioeng Biotechnol. 2015 Aug 27;3:123. doi: 10.3389/fbioe.2015.00123 (PMC4550784; doi:10.3389/fbioe.2015.00123)
Supplement: Supplementary file 1 [file Table_1.PDF]

Supporting information

Table 1: Identification of *S. acidocaldarius* EPS proteins using nanoRSLC Orbitrap LC-MS/MS. EPS was isolated from *S. acidocaldarius* unsaturated biofilms (4 d, 78°C) and digested with trypsin. Proteins were categorized according to arCOG functional codes and annotations using the updated genome information (Esser et al., 2011). Subcellular localizations were performed with PSORTb (Yu et al., 2010) Gene IDs marked in bold represent proteins identified in *S. acidocaldarus* membrane vesicles (Ellen et al., 2009).

Gene ID	arCOG assignment	arCOG functional code	arCOG annotation	Subcellular localization
Saci_0243	arCOG04237	C	Citrate synthase	Cytoplasmic
Saci_0315	arCOG00448	C	Electron transfer flavoprotein, alpha and beta subunits	Cytoplasmic
Saci_0916	arCOG01926	C	Aerobic-type carbon monoxide dehydrogenase, middle subunit CoxM/CutM homolog	Cytoplasmic
Saci_1009	arCOG01167	C	Aerobic-type carbon monoxide dehydrogenase, large subunit CoxL/CutL homolog	Cytoplasmic
Saci_1020	arCOG01167	C	Aerobic-type carbon monoxide dehydrogenase, large subunit CoxL/CutL homolog	Cytoplasmic
Saci_1214	arCOG01697	C	Aconitase A (Uhrigshardt et al., 2001)	Cytoplasmic
Saci_1265	arCOG01337	C	Succinyl-CoA synthetase, beta subunit	Unknown
Saci_1742	arCOG01167	C	Aerobic-type carbon monoxide dehydrogenase, large subunit CoxL/CutL homolog	Unknown
Saci_2117	arCOG01167	C	Aerobic-type carbon monoxide dehydrogenase, large subunit CoxL/CutL homolog	Unknown
Saci_2154	arCOG00853	C	Malic enzyme	Cytoplasmic
Saci_2269	arCOG01926	C	Aerobic-type carbon monoxide dehydrogenase, middle subunit CoxM/CutM homolog	Cytoplasmic
**Saci_2271**	arCOG01167	C	Aerobic-type carbon monoxide dehydrogenase, large subunit CoxL/CutL homolog	Cytoplasmic
Saci_0227	arCOG01252	CH	Lactaldehyde dehydrogenase, NAD-dependent aldehyde dehydrogenase	Cytoplasmic
Saci_1738	arCOG01252	CH	Lactaldehyde dehydrogenase, NAD-dependent aldehyde dehydrogenase	Cytoplasmic
Saci_1857	arCOG01252	CH	Lactaldehyde dehydrogenase, NAD-dependent aldehyde dehydrogenase	Cytoplasmic
Saci_0253	arCOG01698	E	Homoaconitate hydratase/3-isopropylmalate dehydratase large subunit family protein	Cytoplasmic
Saci_0372	arCOG02969	E	Aminopeptidase N	Cytoplasmic
Saci_0798	arCOG01678	E	Archaeal S-adenosylmethionine synthetase	Cytoplasmic

Gene ID	arCOG assignment	arCOG functional code	arCOG annotation	Subcellular localization
Saci_0827	arCOG01877	E	Methionine synthase II (cobalamin-independent)	Unknown
Saci_0828	arCOG01876	E	Methionine synthase II (cobalamin-independent)	Cytoplasmic
Saci_1358	arCOG00070	E	Glycine/serine hydroxymethyltransferase	Cytoplasmic
Saci_1412	arCOG00861	E	Aspartokinase	Cytoplasmic
Saci_1428	arCOG01130	E	Aspartate/tyrosine/aromatic aminotransferase	Cytoplasmic
Saci_1483	arCOG01909	E	Glutamine synthetase (Yin et al., 1998)	Cytoplasmic
Saci_1617	arCOG00112	E	Argininosuccinate synthase	Unknown
Saci_1620	arCOG01594	EF	Carbamoylphosphate synthase large subunit	Cytoplasmic
Saci_1715	arCOG04045	EG	Dihydroxyacid dehydratase/phosphogluconate dehydratase	Unknown
Saci_2255	arCOG02001	EH	Acetolactate synthase large subunit or other thiamine pyrophosphate-requiring enzyme	Cytoplasmic
Saci_2281	arCOG03657	EH	Acetolactate synthase large subunit or other thiamine pyrophosphate-requiring enzyme	Cytoplasmic
Saci_1079	arCOG01459	ER	Threonine dehydrogenase or related Zn-dependent dehydrogenase	Cytoplasmic
Saci_0707	arCOG04346	F	5-formaminoimidazole-4-carboxamide-1-beta-D-ribofuranosyl 5'-monophosphate synthetase (purine biosynthesis)	Unknown
Saci_0789	arCOG04048	F	Deoxycytidine deaminase	Unknown
Saci_0671	arCOG04180	G	Archaeal fructose 1,6-bisphosphatase	Cytoplasmic
Saci_0806	arCOG00767	G	Phosphomannomutase	Cytoplasmic
**Saci_0854**	arCOG00574	G	Ribulose 1,5-bisphosphate synthetase, converts PRPP to RuBP, flavoprotein	Cytoplasmic
Saci_1356	arCOG00493	G	Glyceraldehyde-3-phosphate dehydrogenase/erythrose-4-phosphate dehydrogenase	Cytoplasmic
Saci_0059	arCOG04435	GC	Phosphoenolpyruvate carboxylase	Cytoplasmic
Saci_0396	arCOG00977	H	Precorrin-6B methylase 2	Cytoplasmic
Saci_0646	arCOG04137	H	S-adenosylhomocysteine hydrolase	Cytoplasmic
Saci_0720	arCOG00972	H	Nicotinamide mononucleotide adenylyltransferase	Cytoplasmic
Saci_1555	arCOG04075	H	Pyridoxine biosynthesis enzyme	Cytoplasmic
**Saci_0306**	arCOG04201	I	Acyl-coenzyme A synthetase/AMP-(fatty) acid ligase	Cytoplasmic
Saci_1033	arCOG01707	I	Acyl-CoA dehydrogenase	Cytoplasmic
Saci_1071	arCOG05965	I	Acyl-CoA dehydrogenase	Cytoplasmic
Saci_1123	arCOG01707	I	Acyl-CoA dehydrogenase	Cytoplasmic
Saci_2232	arCOG01280	I	Acetyl-CoA acetyltransferase	Cytoplasmic

Gene ID	arCOG assignment	arCOG functional code	arCOG annotation	Subcellular localization
Saci_2233	arCOG01279	I	Acetyl-CoA acetyltransferase	Cytoplasmic
Saci_2288	arCOG01278	I	Acetyl-CoA acetyltransferase	Cytoplasmic
Saci_1122	arCOG00856	IQ	Acyl-CoA synthetase (AMP-forming)/AMP-acid ligase II	Cytoplasmic
Saci_0199	arCOG01262	IQR	Short-chain alcohol dehydrogenase	Cytoplasmic
Saci_0081	arCOG04239	J	Ribosomal protein S4 or related protein	Cytoplasmic
Saci_0082	arCOG04240	J	Ribosomal protein S11	Cytoplasmic
Saci_0585	arCOG04093	J	Ribosomal protein S4E	Cytoplasmic
Saci_0596	arCOG04071	J	Ribosomal protein L4	Unknown
Saci_0597	arCOG04070	J	Ribosomal protein L3	Cytoplasmic
Saci_0620	arCOG04186	J	Ribosomal protein S3AE	Cytoplasmic
**Saci_0685**	arCOG01561	J	Translation elongation factor EF-1alpha (GTPase)	Cytoplasmic
Saci_0758	arCOG04154	J	Ribosomal protein S8E	Cytoplasmic
Saci_0768	arCOG00406	J	Aspartyl/asparaginyl-tRNA synthetase	Cytoplasmic
Saci_0853	arCOG04182	J	Ribosomal protein S24E	Cytoplasmic
Saci_1261	arCOG00401	J	Threonyl-tRNA synthetase	Cytoplasmic
Saci_1347	arCOG01923	J	Protein implicated in ribosomal biogenesis, Nop56p homolog	Cytoplasmic
Saci_1544	arCOG00810	J	Methionyl-tRNA synthetase	Cytoplasmic
Saci_0692	arCOG04257	K	DNA-directed RNA polymerase subunit A'	Cytoplasmic
Saci_0834	arCOG00675	K	DNA-directed RNA polymerase, subunit E'	Cytoplasmic
Saci_1874	arCOG03482	K	Predicted transcriptional regulator associated with CRISPR system, contains COG1517 family domain	Cytoplasmic
Saci_1507	arCOG00492	KE	Transcriptional regulators containing a DNA-binding HTH domain and an aminotransferase domain (MocR family)	Cytoplasmic
Saci_1629	arCOG04298	L	Predicted adenosine-specific kinase	Cytoplasmic
Saci_1174	arCOG04148	NU	Predicted ATPase involved in biogenesis of archaeal flagella	Cytoplasmic membrane
Saci_0335	arCOG02062	O	Predicted redox protein, regulator of disulfide bond formation	Cytoplasmic
**Saci_1401**	arCOG01257	O	Chaperonin GroEL (HSP60 family)	Cytoplasmic
Saci_2276	arCOG00636	O	Hydrogenase maturation factor	Cytoplasmic
Saci_1103	arCOG02143	Q	Aromatic ring hydroxylase	Cytoplasmic
Saci_2294	arCOG02143	Q	Aromatic ring hydroxylase	Cytoplasmic
**Saci_0331**	arCOG01064	R	NAD(FAD)-dependent dehydrogenase	Cytoplasmic
Saci_0336	arCOG02064	R	Peroxiredoxin family protein	Cytoplasmic
Saci_0998	arCOG00040	R	Predicted phosphoribosyltransferase	Unknown
Saci_2205	arCOG01455	R	Zn-dependent alcohol dehydrogenase	Cytoplasmic
Saci_2230	arCOG01288	R	Predicted nucleic-acid-binding protein containing a Zn-ribbon	Cytoplasmic
Saci_0064	arCOG05888	S	Chromosomal protein Sac7d	Cytoplasmic

Gene ID	arCOG assignment	arCOG functional code	arCOG annotation	Subcellular localization
Saci_0333	arCOG02114	S	Uncharacterized conserved protein	Unknown
Saci_0386	arCOG02738	S	NifX family protein	Cytoplasmic
Saci_1678	arCOG07227	S	Uncharacterized conserved protein	Cytoplasmic
Saci_0274	arCOG00790	V	CRISPR-associated protein Cas4 (RecB family exonuclease)	Cytoplasmic

Figure 1

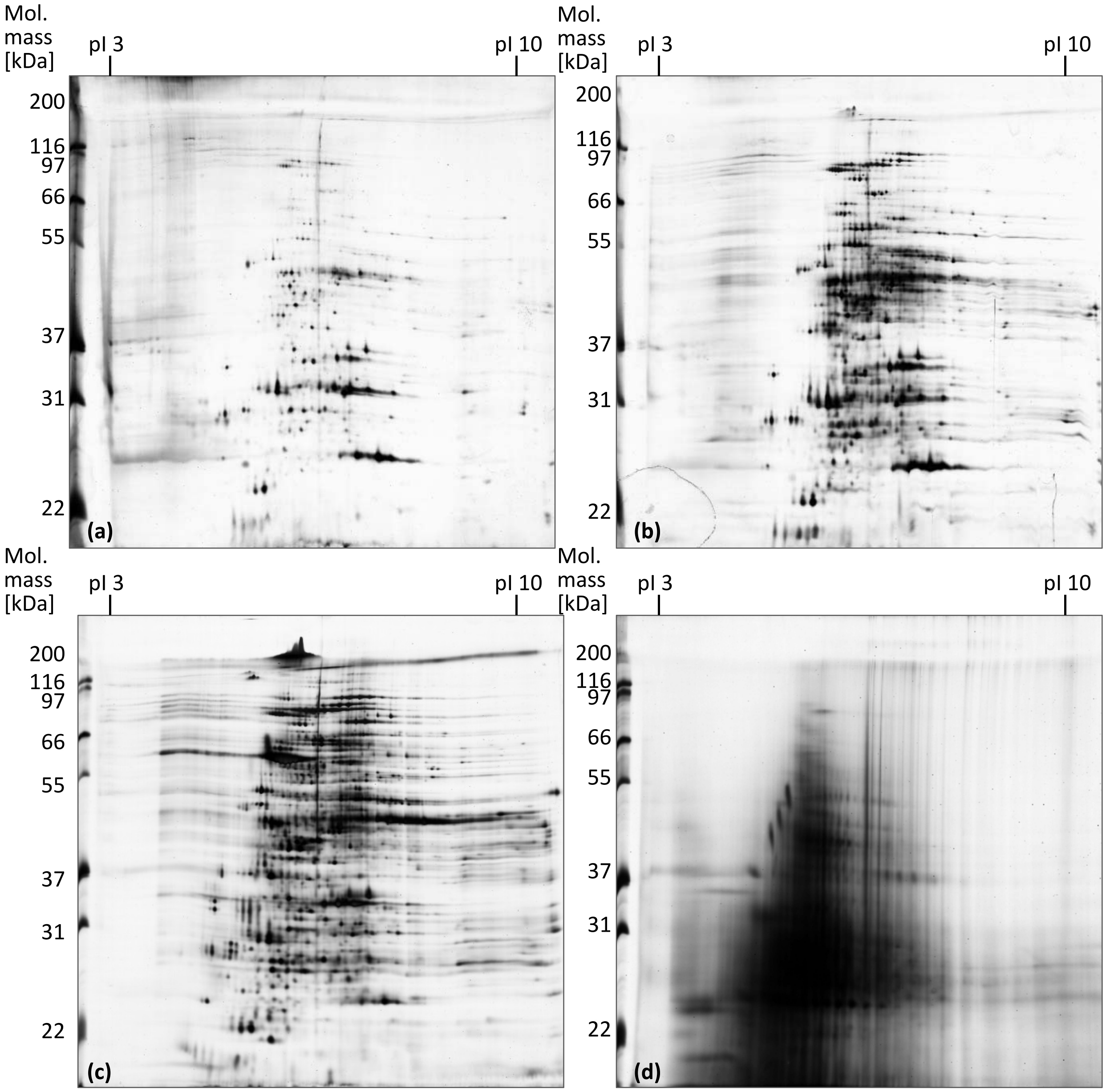

Figure 1. Two dimensional gel electrophoresis of EPS proteins of *S. acidocaldarius* isolated with (a) shaking, (b) CER, (c) crown ether and (d) NaOH. 200 µg EPS protein were applied for IEF (linear IPG strips). Polyacrylamide gels were stained with silver according to Blum *et al.* (1987). Molecular mass marker: PageRuler Prestained Protein Ladder (Fermentas).

References:

Ellen, A.F., Albers, S.V., Huibers, W., Pitcher, A., Hobel, C.F., Schwarz, H., Folea, M., Schouten, S., Boekema, E.J., Poolman, B., and Driessen, A.J. (2009). Proteomic analysis of secreted membrane vesicles of archaeal *Sulfolobus* species reveals the presence of endosome sorting complex components. *Extremophiles* 13**,** 67-79.

Esser, D., Kouril, T., Zaparty, M., Sierocinski, P., Chan, P.P., Lowe, T., Van Der Oost, J., Albers, S.V., Schomburg, D., Makarova, K.S., and Siebers, B. (2011). Functional

curation of the *Sulfolobus solfataricus* P2 and *S. acidocaldarius* 98-3 complete genome sequences. *Extremophiles* 15**,** 711-712.

Uhrigshardt, H., Walden, M., John, H., and Anemuller, S. (2001). Purification and characterization of the first archaeal aconitase from the thermoacidophilic *Sulfolobus acidocaldarius*. *Eur. J. Biochem.* 268**,** 1760-1771.

Yin, Z., Purschke, W.G., Schafer, G., and Schmidt, C.L. (1998). The glutamine synthetase from the hyperthermoacidophilic crenarcheon *Sulfolobus acidocaldarius*: isolation, characterization and sequencing of the gene. *Biol. Chem.* 379**,** 1349-1354.

Yu, N.Y., Wagner, J.R., Laird, M.R., Melli, G., Rey, S., Lo, R., Dao, P., Sahinalp, S.C., Ester, M., Foster, L.J., and Brinkman, F.S. (2010). PSORTb 3.0: improved protein subcellular localization prediction with refined localization subcategories and predictive capabilities for all prokaryotes. *Bioinformatics* 26**,** 1608-1615.
